# Erectile dysfunction and associated factors among men with diabetes mellitus from a tertiary diabetic center in Northern Sri Lanka

**DOI:** 10.1186/s13104-019-4244-x

**Published:** 2019-04-05

**Authors:** Balasingam Nisahan, Thirunavukarasu Kumanan, Nadarajah Rajeshkannan, Thampipillai Peranantharajah, Mahalingam Aravinthan

**Affiliations:** 1 Teaching Hospital Jaffna, Jaffna, Sri Lanka; 2 Department of Medicine, Faculty of Medicine, University of Jaffna, Jaffna, Sri Lanka; 3 Civic Park Medical Center, Sydney, NSW 2145 Australia

**Keywords:** Erectile dysfunction, Diabetes, Co existing hypertension

## Abstract

**Objective:**

Prevalence of erectile dysfunction (ED) in diabetic men is considerably high but it is often underdiagnosed and undermanaged. There were no data available about the prevalence and the risk factors of ED in our region. So a cross-sectional study was conducted to identify the prevalence and associated risk factors of ED in a tertiary care diabetic center in Northern Sri Lanka.

**Results:**

326 diabetic male patients between ages 18–60 years were interviewed. Majority (62.9%; 95% CI 57.5–68.0%) of the diabetic patients suffered from ED and 22.4% (95% CI 17.8–26.8%) were found to have severe ED. Most of the patients (98.8%) were not screened or treated for ED. Bivariate analysis showed age above 40, duration of DM (> 5 years), type of diabetes (type 2), having micro-vascular complications, co-existing hypertension, BMI, consuming unsafe level of alcohol and taking beta-blockers were associated with ED at 5% level (P < 0.05). This study failed to show association with dyslipidemia, macro vascular complications such as coronary artery disease (CAD, P-0.052), glycemic control (P-0.082) and smoking. Regression analysis revealed age > 40 (AOR: 2.13; 95% CI 1.05–4.33), duration of diabetes (AOR: 2.90; 95% CI 1.67–5.01), co-existing hypertension (AOR: 1.8; 95% CI 1.06–3.06), and unsafe level alcohol intake (AOR: 3.14; 95% CI 1.76–5.59) were independent risk factors.

**Electronic supplementary material:**

The online version of this article (10.1186/s13104-019-4244-x) contains supplementary material, which is available to authorized users.

## Introduction

Erectile dysfunction is defined as persistent inability to achieve or maintain erection of the penis firm enough to have satisfactory sexual intercourse [1]. Prevalence of ED in diabetic men ranges from 35 to 90%. Erectile dysfunction is two to threefold higher in men with DM compared to men without DM [2]. ED might present in the early stages of diabetes mellitus or sometimes as a chief complaint of diabetic patients [3].

Sexual function is one of the important indices of quality of life. The development of ED is negatively associated with men’s relationship, social interactions, emotional and particularly psychological well-being [4]. Erectile dysfunction is a preventable diabetic complication. Around 95% of patients with erectile dysfunction related to DM can be treated successfully [5].

Hyperglycemia is the main determinant of vascular diabetic related complications. But it is still not clear the involvement of hyperglycaemia in the pathogenesis of sexual dysfunction [6]. Also ED occurs 10–15 years earlier in men with diabetes than non-diabetics [7]. Increased age and duration of diabetes have been associated with an increased risk of ED [8]. There are number of factors contributing for the erectile dysfunction in diabetic men such as hypertension, obesity, dyslipidemia, smoking and autonomic neuropathy [9]. However the intensity of the risk factors could vary from country to country. There were no data available about the prevalence and the risk factors of ED in diabetic men in our region. Owing to the influence of South-Asian socio cultural norms and practices ED is often underdiagnosed in Sri Lanka. This study was designed to identify the prevalence and the risk factors of ED in diabetic men.

## Main text

### Methods

This was a cross-sectional study in patients attending to diabetic center Teaching Hospital Jaffna. All the male diabetic patients between ages 18–60 were recruited during the study period. Patients who are mentally incompetent; suffering from end stage organ failure such as chronic kidney disease, chronic liver cell disease and heart failure; history of spinal injury and who had major stroke were excluded from the study.

Ethical clearance was obtained from ethical review committee, faculty of Medicine, University of Jaffna. Data collected for a period of 5 months from August 2017. We collected details from all male patients who satisfied the inclusion criteria. Data collection done by investigators via interviewer administered questionnaire.

The evaluation of ED was done by International Index of Erectile function (IIEF-5) questionnaire. This widely accepted tool evaluates five aspects of sexual function such as erectile function, orgasm, desire for sex, satisfaction after intercourse and overall satisfaction. Each aspect was evaluated by five points scale and the score more than 21 was considered as normal erectile function. Depending on the score, ED is further classified as mild (17–21), mild to moderate (12–16), moderate (8–11) and severe ED (less than 8) [10].

Measurement of weight and height was done with standard steps to calculate BMI. Diabetic related micro vascular and macro vascular complications, duration of diabetes, history of hypertension, hyperlipidemia and details of hypertensive medications were retrieved from medical records. Latest HbA1C and fasting blood sugar readings were also obtained from records.

Smokers were classified according to standard National Health Interview Survey. In their life time, if patient smoked at least 100 cigarettes is considered as smoker. Safe level of alcohol intake was defined according to the American Heart Association as no more than two standard drinks per day.

Analysis of the data was done by using SPSS version 25. Prevalence was described using percentage with confidence interval (CI). Results were summarized using tables and graphs. Chi squared test was used to establish association. Establishment of statistical significance was done at P < 0.05. Binary Logistic regression analysis was conducted by using significant variables identified in bivariate analysis. Variable(s) entered on into the model: age, type of DM, duration of DM, presence of micro vascular complications, existing-HT, BMI, taking beta-blockers and consuming unsafe level of Alcohol.

### Results

#### Characteristic of study population

The mean age of the 326 male diabetics was 49 ± 7.5 years. Majority (58.6%) belongs to lower socio economic group, 33.1% studied up to primary school and 38% completed secondary school. Among them 33.1% were reported unsafe level of alcohol consumption and 21.8% were smokers.

**Fig. 1:**
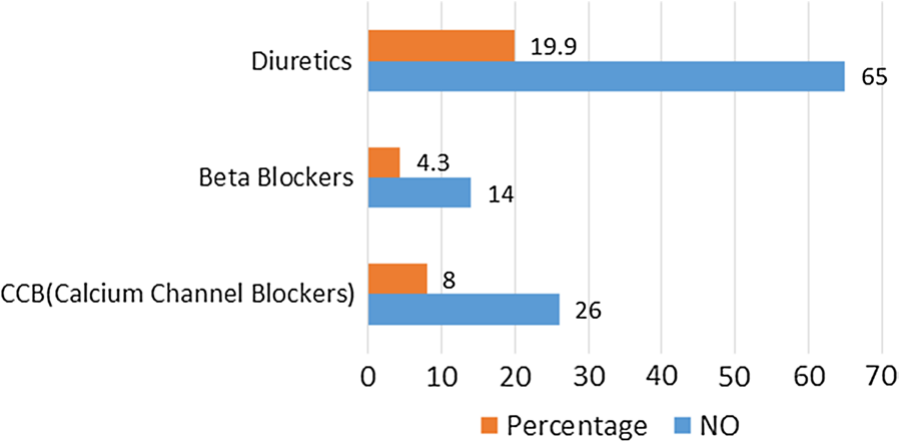
Hypertensive medication usage among participants

Around 56% of participants have been diagnosed within 5 years and 23.6% have diabetes for more than 10 years. Majority of (62.0%) participants’ diabetic control not up to the mark (HbA1C ≥ 7). Significant percentage (45.7%) of participants were identified to having micro vascular complications such as neuropathy (15.3%), nephropathy (25.5%) and retinopathy (22.5%), but only 9.2% were identified as having macro vascular complications such as cerebrovascular accident (CVA), coronary artery disease (CAD) and peripheral vascular disease (PVD). Further 46.6% of study population had co-existing hypertension and 44.5% had hyperlipidemia. Figure 1 shows the hypertensive medication use among the participants.

#### Prevalence of ED among diabetics

**Fig. 2:**
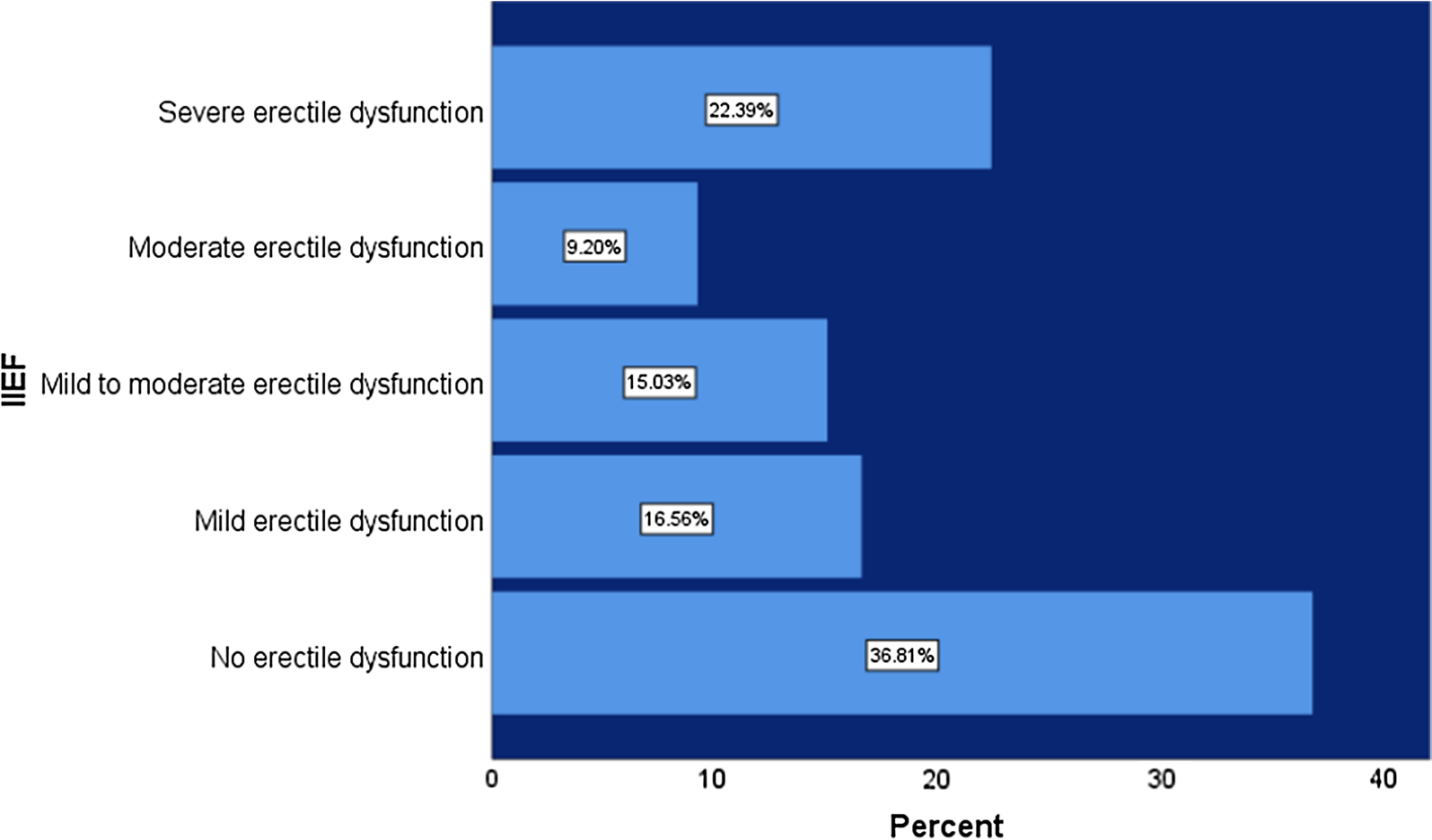
Distribution of severity of erectile dysfunction among study participants

Among participants erectile dysfunction (ED) was identified in 62.9% (CI 57.5–68.0%), while 22% (CI 17.8–26.8%) were found to have severe ED (Fig. 2). Most of the patients who were included in the study (98.8%) were not screened or managed for ED.

#### Associated factors with ED

Bivariate analysis showed association with increasing age (P < 0.0001), duration of DM (P < 0.0001); type of diabetes (type 2) (OR = 3.66; CI 1.22–10.96), associated micro vascular complications such as diabetic neuropathy (OR = 1.33; 95% CI 1.12–1.51, P-0.007) and nephropathy (OR = 1.32; CI 1.12–1.54, P-0.002); co-existing hypertension (OR = 1.39; CI 1.18–1.64, P < 0.005); BMI > 25 (P = 0.035, OR: 1.74; CI 1.09–2.79), consuming unsafe level of alcohol (OR = 1.34; CI 1.14–1.56) and taking beta-blockers (OR = 1.50; CI 1.27–1.78; P-0.019) (Table 1). But our data failed to show association with socio demographic variables such as family income (P-0.124) and education (P-0.069), co-existing conditions such as dyslipidaemia (P = 0.43), diabetic retinopathy (P-0.332), macro-vascular complications such as CAD (P = 0.052), PVD (p = 0.72) and also between ED and control of diabetes (HbA1C < 7) (P = 0.082) as well as smoking and ED (P = 0.153).

**Table 1:** Association between selected factors with erectile dysfunction

Characteristics (variables)	Status of erectile dysfunction			Level of significance and odds ratio with 95% CI
	Presence	Absence	Total	
Age above 40**	187 (57.4%)	87 (26.7%)	274 (84.0%)	P < 0.0001, OR: 3.73 (CI 2.01–6.93)
Type of diabetes**				P-0.014, OR: 3.66 (CI 1.22–10.96)
Type 1	5 (1.5%)	10 (3.1%)	15 (4.6%)	
Type 2	201 (61.6%)	110 (33.7%)	311 (95.4%)	
Duration of diabetes** > 5 years	111 (34.0%)	31 (9.5%)	142 (43.6%)	P < 0.0001, OR: 3.35 (CI 2.05–5.49)
Poor diabetic control (HbA1C ≥ 7)	135 (41.4%)	67 (20.6%)	202 (62.0%)	P-0.082, OR: 1.50 (CI 0.95–2.38)
Unsafe level of alcohol consumption**	82 (25.2%)	26 (8.0%)	108 (33.1%)	P-0.001, OR: 1.34 (CI 1.14–1.56)
Smoker	50 (15.3%)	21 (6.4%)	71 (21.8%)	P-0.153, OR: 0.66 (CI 0.38–1.17)
With micro-vascular complications**	105 (32.2%)	44 (13.5%)	149 (45.7%)	P-0.012, OR: 1.80 (CI 1.11–2.85)
With diabetic neuropathy**	40 (12.3%)	10 (3.1%)	50 (15.3%)	P-0.007, OR: 1.33 (CI 1.12–1.57)
With diabetic nephropathy**	64 (19.6%)	19 (5.8%)	83 (25.5%)	P-0.002, OR: 1.32 (CI 1.13–1.54)
With retinopathy	49 (15.0%)	23 (7.1%)	72 (22.1%)	P-0.332, OR: 0.76 (CI 0.44–1.33)
With macro vascular complications	23 (7.1%)	7 (2.2%)	30 (9.2%)	P-0.108, OR: 2.03 (CI 0.843–4.88)
Co-existing hypertension**	113 (34.7%)	39 (12.0%)	152 (46.6%)	P < 0.0001, OR: 1.39 (CI 1.18–1.64)
Hyperlipidaemia	95 (29.1%)	50 (15.3%)	145 (44.5%)	P < 0.436, OR: 1.07 (CI 0.91–1.26)
Taking beta-blockers**	13 (4.0%)	1 (0.3%)	14 (4.3%)	P = 0.019, OR: 1.50 (CI 1.27–1.78)
BMI ≥ 25**	94 (28.8%)	39 (12.0%)	133 (40.8%)	P = 0.035, OR: 1.74 (CI 1.09–2.79)

**Significant factors

Multivariate logistic analysis revealed age above 40 (AOR: 2.13; CI 1.05–4.33), duration of diabetes (more than 5 years) (AOR: 2.90; CI 1.67–5.02), co-existing hypertension (AOR: 1.80; CI 1.07–3.06), and unsafe level alcohol intake (AOR: 3.14; CI 1.76–5.59) were independent risk factors (Additional file 1: Table S1).

### Discussion

Erectile dysfunction is prevalent among 62.9% of male diabetic patients. Almost similar percentage (69.9%) noted in the research done in Northern Ethiopia recently [11]. The ED among diabetics were reported in previous studies varies between 35 and 90% [12, 13]. The prevalence of severe ED is identified in 22.1% of our population whereas, it is around 5.2% in the study done in Northern Ethiopia [11]. This varying degree of prevalence and severity could be due to the different study population which might have additional under reported risk factors.

Only four patients were getting treatment for ED at the time of interview. Remaining 98.8% were either reluctant to get treatment or unaware of ED treatment modalities. This might be due to the social stigma or not-considering ED as a treatable disease. Therefore, it is obvious that ED is a common worrisome complication of diabetes which is under diagnosed. This could affect the quality of life of the affected men and that might in turn worsen the diabetic control [14].

Increasing age is a common risk factor for ED. In our study even though analysis done in patients between 18 and 60 years prevalence of ED significantly increased with age as in par with some other studies [15]. There are some controversial results in some studies where increasing age has not been shown to be an independent risk factor for ED in diabetes [14]. The duration of DM (> 5 years) is an independent risk factor for ED in our study population (P < 0.001). This finding is similar to shown in other studies [14, 15].

When considering complications of diabetes it is reasonable to assume that both micro vascular and macro vascular complications of diabetes are associated with high risk of ED [16–18]. This study has found that diabetic neuropathy and nephropathy were significantly associated with high risk of ED (P-0.007 and 0.002 respectively) but retinopathy did not show an association (P-0.332). This observation is contradicts to the previous studies and could be explained by a genetic predisposition but need detail evaluation. We could not able to comment on autonomic neuropathy as only a single participant had autonomic neuropathy symptom. Most of the studies identified autonomic neuropathy as a significant risk factor for ED [11, 14]. Macro vascular diseases such as CAD (P- = 0.052), ischemic stroke and PVD did not show significant association with ED in the study population. This may be due to the fact that the age of onset of ischemic stroke and PVD is quite late in this population. Even though CAD relatively common in young age but the association between CAD and ED not established (P-0.052).

Co-existing hypertension is an important cardiovascular risk factor of diabetes showed significant association (P < 0.001) and has to be optimized in order to prevent ED in diabetics. Having hypertension with the back ground of diabetes significantly increases the risk of arthrosclerosis which might affect the penile arteries [7, 8]. Along with this study the association between dyslipidemia and ED is not established [11, 14]. However, a study done in Italy revealed significant relationship between atherogenic dyslipidemia and ED [19].

Recent control of diabetes mellitus showed no significant relationship with ED (P-0.08). Similar finding were also noted in some of the recent studies [7, 11]. However a Nigerian study concluded that poor glycemic control is the most prominent independent risk factor for ED [14]. Therefore serial HbA1c levels over a period could be an important tool in assessing long term glycemic control and would have a better correlation than a single HbA1C value.

Cigarette smoking is a recognized risk for ED in diabetics [8]. However this study failed to show an association with smoking (P-0.153). Similar findings were observed in studies done at different parts of the world [11, 14]. It could be due to the reduced level of smoking habit because of high level of health education they received coupled with high rate of tax imposed up on cigarettes by government. Unsafe amount of alcohol intake was found to be an independent risk factor for ED (OR: 3.14; CI 1.76–5.59). This fact was not evaluated in most of the studies done in other parts of the world and we suggest a further study with the degree of alcohol taken into account.

When considering the medications, significant association with the beta-blockers (P = 0.02) was noted. Other hypertensive medications failed to show association (diuretics; P-0.242 and CCB; P-0.506). Previous studies demonstrated that beta blocker and diuretics are risk factors for ED [20, 21]. Over weight showed an association in bivariate analysis (P = 0.035) but multivariate analysis revealed no association. An association was documented in a study done in India [22]. But the results are controversial in some other studies [12, 14]. Finally family income (P-0.124) did not show an association with ED in contrast a study done in Ethiopia showed an association with lower income [11].

In conclusion, the prevalence of erectile dysfunction is high among diabetic patients and most of them (98.8%) were not screened or managed for ED. Majority of men could be considered as silent suffers. The main predictors of ED were age above 40, duration of DM, hypertension and unsafe level of alcohol intake. Therefore, it is vital to have the routine screening for ED in diabetic clinics periodically and manage accordingly. The co-existing hypertension should be treated with appropriate medications which minimally interfere with sexual dysfunction and preventive measures need to be targeted to reduce the alcohol intake.

## Limitation

The study population slightly different from the diabetic men who attend other clinics as usually patients with complex medical problems tend to attend tertiary Centre.

The evaluation of ED was done by IIEF-5 questionnaire and it is not validated to our population as a result there is a chance misclassification error even though this tool widely accepted worldwide.

We have excluded the patients with major psychiatric illness but psychological evaluation should be carried out, in addition to BMI data could be collected as continuous variable and sample size could be calculated to include possible risk factors to get more accurate results.

A detailed evaluation of the long term glycemic control, lipid profile and smoking could improve the validity of the study.

## Additional file

**Additional file 1: Table S1.** Multivariate logistic regression results of study variables for ED.

## Abbreviations

ED: erectile dysfunction
DM: diabetes mellitus
BMI: body mass index
CI: confidence interval
CAD: coronary artery disease
PVD: peripheral vascular disease
AOR: adjusted odds ratio
IIEF: International Index of Erectile Function
