# Supplementary material for: Erectile dysfunction and associated factors among men with diabetes mellitus from a tertiary diabetic center in Northern Sri Lanka
Source: BMC Res Notes. 2019 Apr 5;12:210. doi: 10.1186/s13104-019-4244-x (PMC6451292; doi:10.1186/s13104-019-4244-x)
Supplement: Supplementary file 1 — Additional file 1: Table S1. Multivariate logistic regression results of study variables for ED. [file 13104_2019_4244_MOESM1_ESM.docx]

**Table S1: Multivariate logistic regression results of study variables for ED**

|  | | | | | | | | |
| --- | --- | --- | --- | --- | --- | --- | --- | --- |
| Variable | | B | S.E (Standard error) | Significance | Exp (B) | 95% C.I. for EXP(B) | |  |
|  |  |  |  |  |  | Lower | Upper |  |
|  | Age above | .756 | .362 | .037 | 2.131 | 1.048 | 4.334 |  |
|  | Type 2 DM | 1.178 | .691 | .088 | 3.248 | .838 | 12.588 |  |
|  | Alcohol Unsafe-level | 1.144 | .294 | .000 | 3.139 | 1.764 | 5.587 |  |
|  | Taking Beta Blocker | 1.622 | 1.166 | .164 | 5.061 | .515 | 49.734 |  |
|  | BMI 25 an above | .349 | .266 | .190 | 1.418 | .841 | 2.389 |  |
|  | Duration >5years | 1.063 | .280 | .000 | 2.895 | 1.672 | 5.012 |  |
|  | Coexisting HT | .590 | .269 | .028 | 1.805 | 1.065 | 3.056 |  |
